# Exploring Metabolic Disruption and Redox Modulation by Senna Leaf Extracts Induces Mortality in the Zoonotic Parasite *Hymenolepis diminuta*

**DOI:** 10.1155/japr/2876272

**Published:** 2025-12-17

**Authors:** Saptarshi Roy, Larisha M. Lyndem

**Affiliations:** ^1^ Parasitology Research Laboratory, Department of Zoology, Visva-Bharati University, Santiniketan, West Bengal, India, visvabharati.ac.in

**Keywords:** anthelminthic, cestode, energy metabolism, glycogen phosphorylase, glycolytic enzyme

## Abstract

Senna leaf extracts exhibit strong anthelmintic effects against the zoonotic cestode *Hymenolepis diminuta*, inducing profound morphological and biochemical alteration. This study investigated the underlying mechanisms, focusing on glycolytic disruption and reactive nitrogen species (RNS) generation. Live *H. diminuta* were exposed in vitro to leaf extracts from *Senna alata*, *Senna alexandrina*, and *Senna occidentalis* (40 mg/mL), with praziquantel as a positive control. Biochemical assays demonstrated that there is a significant alteration in energy metabolism. Glycogen phosphorylase (GPase) activity increased, whereas glycogen synthase (GSase) activity declined, indicating enhanced glycogen catabolism. Parasites accumulated glucose and lactate but exhibited reduced pyruvate and malate, suggesting a shift towards anaerobic metabolism. Key glycolytic enzymes, including pyruvate kinase, phosphofructokinase, phosphoenolpyruvate carboxykinase, and malate dehydrogenase were inhibited, whereas lactate dehydrogenase and glutamate dehydrogenase activities were elevated. Histochemical analysis corroborated these enzymatic changes, demonstrating mitochondrial stress and redox imbalance. Notably, nitric oxide synthase (NOS) activity and nitric oxide (NO) levels were significantly elevated, indicating activation of the NO/cGMP signaling pathway. The resulting oxidative stress disrupted calcium homeostasis and induced flaccid paralysis. Collectively, our results indicate that senna leaf extracts compromise parasite viability by interfering with glycolytic metabolism and promoting RNS generation, underscoring their potential as effective plant‐derived anthelmintic agents.

## 1. Introduction

Helminths, particularly those inhabiting anaerobic and nutrient‐variable environments, depend on efficient metabolic adaptations for survival within their hosts [1, 2]. Among these adaptations, glycogen serves as the principal energy reserve, particularly in cestodes such as *Hymenolepis diminuta* [3]. In the absence of a consistent oxygen supply and under fluctuating glucose availability, these parasites depend on stored glycogen as a major energy source to meet their energy demands [4, 5]. The breakdown and synthesis of glycogen are tightly regulated by key enzymes, including glycogen phosphorylase (GPase), which catalyzes glycogenolysis and glycogen synthase (GSase), which facilitates glycogenesis [6–9]. In helminths, glycolysis is a vital pathway regulated by enzymes such as hexokinase (HK), phosphofructokinase (PFK), and pyruvate kinase (PK), which collectively help generate ATP [10–14]. Additionally, the conversion of pyruvate to lactate is mediated by the enzyme lactate dehydrogenase (LDH). This reaction enables continued energy production in anaerobic environments by regenerating NAD^+^ [14]. Beyond glycolysis, the hexose monophosphate (HMP) pathway plays a significant role in maintaining cellular redox balance and providing biosynthetic precursors [15]. This pathway produces NADPH, which is essential for fatty acid synthesis and for generating ribose sugars, both of which are crucial for nucleic acid synthesis and overall parasite survival [12]. However, under stress conditions, such as host immune responses or exposure to antiparasitic agents, the parasite′s metabolic homeostasis can be severely disrupted [16, 17]. This dysregulation can cause overproduction of reactive nitrogen species, including nitric oxide (NO), resulting in cytotoxic and neurotoxic effects on parasites [17, 18]. The correlation between disrupted energy metabolism and elevated RNS generation is particularly associated with oxidative stress, redox imbalance, lipid peroxidation, and DNA damage [18, 19]. Furthermore, NO plays a critical signaling role in helminth neuromuscular coordination. Overproduction of NO, often mediated by inducible nitric oxide synthase (iNOS), can impair neuromuscular function, leading to paralysis and eventual death of the parasite [20, 21]. Moreover, NO disrupts calcium homeostasis and interferes with neurotransmission, making it a potent mediator for disrupting parasitic survival. Consequently, pathways regulate NO synthesis and represent novel targets for anthelmintic drug development. Considering growing resistance to conventional anthelmintic drugs such as praziquantel (PZQ) and albendazole, there is an urgent need to explore alternative, sustainable therapeutic strategies [22–24]. However, the precise mechanisms by which senna extracts affect helminth metabolism and signaling pathways remain poorly understood. Medicinal plant–derived natural products are increasingly investigated for their antiparasitic properties. [25–27]. Among medicinal plants, species of the *Senna* genus including *Senna alata, Senna alexandrina*, and *Senna occidentalis* have shown considerable anthelmintic potential. Our previous research demonstrated that crude extracts from these Senna plant leaf extracts cause extensive morphological damage to *H. diminuta*, affecting the tegument, musculature, calcium homeostasis, and mitochondrial biogenesis [28–31]. Building on these findings, the present study investigates the mechanisms by which senna extracts disrupt parasite physiology, with a focus on energy metabolism. Particular attention is given to the NO‐cGMP signaling pathway, a key regulator of neuromuscular function. Considering that fact, we propose that phytochemical constituents in Senna leaves impair metabolic homeostasis and enhance RNS production, ultimately compromising parasite viability. By targeting both metabolic and neuromuscular systems, Senna leaf extracts represent a promising plant‐based alternative for combating helminth infections.

## 2. Materials and Methods

### 2.1. Collection and Identification of the Plants

Every year in November, during the flowering season, fresh young leaves of *S. alata* (L.) Roxb. (SAL), *S*. *occidentalis* (L.) Link. (SOC), and S. *alexandrina* Mill. (SAX) were collected from our university campus to prepare leaf extracts. The plant material used in this study was examined for taxonomic identification of the herbarium specimen, and was carried out by Dr. Adani Lokho, Professor at Visva‐Bharati University, Santiniketan, India. The authenticated herbarium specimen has been deposited at the Central National Herbarium, Kolkata, West Bengal, India, under Voucher Specimen Numbers VBSL‐1, VBSL‐2, and VBSL‐3 for *S. alata, S. occidentalis,* and *S. alexandrina*, respectively, where it is maintained under the care of the scientific in‐charge for public reference [28–31].

### 2.2. Preparation of Ethanolic Leaf Extracts

Fresh leaves from the three *Senna* species were collected, and leaf extracts were prepared following the method of Kundu et al. [31]. In brief, senna leaves were sunshade dried, crushed, and ground into a powder. About 250 g of this powdered leaf was added to 1 L of 90% ethanol and left for 8 h to run in the Soxhlet Extraction Apparatus from Borosil (Part Number: FX‐3840030). The slurry was filtered via a Whatman filter paper, and the filtrate was concentrated under reduced pressure in order to create a semisolid mass by using a rotary evaporator (EYELA Rotary evaporator, model: N‐1110V‐W) and stored at 4°C for further use.

### 2.3. Maintenance of *H. diminuta* in Rat Model

The life cycle of *H. diminuta* was maintained using Swiss albino rats (Sprague–Dawley Spartan) as the definitive host and *Tribolium* sp. beetles as the intermediate host. Swiss albino rats (8 weeks old, 150–200 g) were infected orally with ~10 cysticercoids in suspension. All experiment protocols were approved by the University Institutional Animal Ethics Committee (IAEC) (Protocol No. IAEC/VB/2017). All the Swiss albino rats were purchased from a registered animal provider (Regd. No: 1828/PO/B1/8/15/CPCSEA) for this study. All experimental animals were maintained in the Animal House Facility, Department of Zoology, Visva‐Bharati University, under IAEC‐approved conditions. Rats were housed five per polycarbonate cage with autoclaved corn cob bedding, provided a 12‐h light/dark cycle, and given ad libitum access to standard rodent diet and sterile drinking water.

### 2.4. Collection of *H. diminuta* From Swiss Albino Rats

Infected rats containing adult *H. diminuta* in the small intestine were euthanized using carbon dioxide (CO_2_) inhalation, following the University IAEC guideline and in compliance with the recommendations of the American Veterinary Medical Association (AVMA) Guidelines. CO_2_ was introduced at a controlled rate of 20%–30% chamber volume per minute to minimize distress. The animals were monitored continuously, and death was confirmed by the absence of respiration and heartbeat, followed by cervical dislocation. After euthanasia, the animals were dissected, and intestinal parasites were carefully recovered for further in vitro experiments.

### 2.5. In Vitro Experimental Design

Live parasitic worms were collected from the intestines of euthanized rats. Twenty Swiss albino rats were used across five independent experiments, divided into four groups of five, with an infection success rate of approximately 70%. From each infected rat, 4–6 intact parasites were recovered. For in vitro assays, ~2 g of worms (about five parasites) were individually exposed to 40 mg/mL of each senna leaf extract, a concentration chosen based on preliminary dose response experiments demonstrating maximal anthelmintic activity without nonspecific toxicity and supported by prior studies [28, 29, 31, 32]. PZQ (5 *μ*g/mL) served as a positive control, whereas negative controls were maintained in PBS. Treatments were conducted at 37^°^C ± 5^°^C, with paralysis marking the endpoint. The parasites were collected at the point of paralysis for biochemical and histochemical assays (Table S1).

### 2.6. Biochemical Estimation

#### 2.6.1. Estimation of Glycogen

Alkali‐soluble glycogen was estimated using the anthrone reagent method described by Steifer et al. [33]. Briefly, approximately 100 mg of wet weight from treated and control parasite tissues was digested in 3 mL of 30% (w/v) KOH at 70°C for 20 min. After cooling, 0.2 mL of saturated Na_2_SO_4_ and 5 mL of 95% ethanol were added, followed by centrifugation at 10,000 g for 10 min at 25°C to precipitate glycogen, repeated three times. Glycogen was then quantified using 0.2% (w/v) anthrone in concentrated H_2_SO_4_, and absorbance was measured at 620 nm using a UV‐Vis spectrophotometer. Concentrations were calculated using a standard curve.

#### 2.6.2. Estimation of Different Metabolites

Glycolytic metabolites were estimated using the enzymatic method of Bergmeyer [34]. Parasites were homogenized (10% w/v) in 50 mM Tris–HCl buffer (pH 7.4), and cellular proteins were precipitated by using 2 M perchloric acid. Followed by centrifugation at 10,000 g for 10 min, the supernatant was neutralized with 2 M NaOH and used for measuring metabolites. Metabolites were assayed using specific reaction mixtures. Glucose was measured in triethanolamine buffer (pH 7.6) containing MgCl_2_, NADP^+^, ATP, HK, and glucose‐6‐phosphate dehydrogenase (G6PDH). Pyruvate was assayed in Tris–HCl (pH 7.4) with NADH and LDH. L‐lactate estimation included glycine, hydrazine hydrate, NAD^+^, and LDH. Malate was measured in Tris–HCl (pH 7.4) with glutamate, NAD^+^, malate dehydrogenase (MDH), and glutamate oxaloacetate transaminase. Each mixture received 100 *μ*L extract supernatant and was incubated at 38°C for 30 min to measure glucose, pyruvate and malate, whereas 2 h for lactate. Absorbance was recorded at 340 nm, and metabolite concentrations were calculated using the molar extinction coefficient of 6.22 × 10^6^ M·cm for NADH/NADPH.

#### 2.6.3. Estimation of GPase and GSase Activity

##### 2.6.3.1. Preparation of Tissue Sample

A 10% (w/v) tissue homogenate of the treated and control parasites was prepared following the method of Russel and Storey [35] in homogenizing buffer, containing imidazole–HCl buffer (20 mM, pH 7.2), NaF (100 mM), EDTA (10 mM), EGTA (10 mM), 2‐mercaptoethanol (15 mM), and PMSF (0.1 mM). The homogenate was centrifuged at 10,000 g at 4°C for 10 min, and the supernatant was used for the enzyme assays.

GPase activity was assayed following the method of Moon et al. [36]. The reaction mixture of 1 mL contained 60 mM potassium phosphate buffer (pH 7.2), NADP^+^ (0.5 mM), glucose 1,6‐bisphosphate (5 mM), AMP (2.5 mM), phosphoglucomutase (5 units), G6PDH (5 units), glycogen (10 mg), and tissue extract (100 *μ*L). For GSase activity, it was assayed following the method of Schwartz et al. [37]. The reaction mixture contained imidazole–HCl buffer (60 mM, pH 7.5), phosphoenolpyruvate (5 mM), UDP‐glucose (6 mM), NADH (0.15 mM), KCl (150 mM), MgCl_2_ (15 mM), glycogen (2 mg), PK (10 units), LDH (10 units), and, 5 *μ*mol glucose 6‐phosphate. A 100‐*μ*L sample of the tissue extract was added to the preincubated assay mixture at 38°C for 10 min.

#### 2.6.4. Glycolytic Enzyme Assay

##### 2.6.4.1. Tissue Processing

To assay key regulatory glycolytic and carbohydrate metabolism enzymes, tissue processing followed the method of Das et al. [38]. Frozen tissues were thawed on ice, and 10% (w/v) homogenates were prepared in buffer containing 50 mM Tris–HCl (pH 7.4), 0.3 M sucrose, 1 mM EDTA, 2 mM MgCl_2_, and 3 mM 2‐mercaptoethanol. The homogenates were treated with 0.5% Triton X‐100 (1:1) for 30 min, followed by 30 s of sonication (Soniprep 150) to disrupt mitochondria. The samples were then centrifuged at 10,000 g for 15 min at 4°C, and the supernatant was used for enzyme assays.

##### 2.6.4.2. Subcellular Fraction

Mitochondrial and cytosolic fractions were prepared by following the methods of Das et al. [38]. The differential centrifugation of a 10% homogenate of the parasite tissue in a fractionating buffer containing 50 mM Tris–HCl (pH 7.4), 0.3 M sucrose, 1 mM EDTA, 2 mM MgCl_2_, and 3 mM 2‐mercaptoethanol was performed. LDH was used as the cytosolic marker and glutamate dehydrogenase (GDH) (L‐glutamate: NAD(P) + oxidoreductase; EC 1.4.1.3] as the mitochondrial marker to assess the complete separation process of the different subcellular fractions.

##### 2.6.4.3. Estimation of Enzymes Activity

The activities of key enzymes involved in glycolysis and carbohydrate metabolism were determined spectrophotometrically using standard procedures. HK was assayed following Bergmeyer [34] using a 1‐mL reaction mixture containing Tris–HCl buffer (pH 7.4), D‐glucose, NADP^+^, ATP, MgCl_2_, G6PDH, and tissue extract. PFK activity was measured by the modified method of Buckwitz et al. [39] using Tris–HCl buffer (pH 7.2), fructose‐6‐phosphate, NADH, ATP, MgCl_2_, KCl, K_2_HPO_4_, aldolase, triosephosphate isomerase, glycerophosphate dehydrogenase, and extract. Phosphoenolpyruvate carboxykinase (PEPCK) was assayed following Mommsen and Moon [40] with Tris–HCl buffer (pH 7.4), phosphoenolpyruvate, NADH, GDP, NaHCO_3_, MnCl_2_, MDH, and extract. PK activity was determined using the method of Bucher and Saz [41], with imidazole buffer (pH 7.6), phosphoenolpyruvate, NADH, ADP, KCl, MgSO_4_, LDH, and extract. LDH and MDH activities were estimated according to Vorhaben [42] and Kun and Volfin [43], respectively. Malic enzyme (ME) activity followed the Bergmeyer method [34], using Tris–HCl, pyruvate, NADPH, and NaHCO_3_. Pyruvate carboxylase (PC) activity was estimated by the Mommsen and Moon [40] method using Tris–HCl (pH 7.8), acetyl CoA, NaHCO_3_, pyruvate, NADH, MgCl_2_, MDH, and extract. G6PDH activity was estimated using the DeMoss et al. method [44] with Tris–HCl, MgCl_2_, NADP^+^, and glucose‐6‐phosphate. GDH activity was measured using the Olson and Anfinsen method [45], with potassium phosphate buffer (pH 8.5), ammonium chloride, *α*‐ketoglutarate, NADH, and EDTA. All assays used 100 *μ*L of tissue extract. Reaction mixtures were preincubated at 38°C for 5 min, and the reaction was initiated by adding the extract. Optical density was monitored at 340 nm every 10 s for 3 min using a UV–Vis spectrophotometer. Specific enzyme activities were expressed as units/mg protein concentration, and protein concentration was measured by the Lowry et al. method [46]. Enzyme activities are expressed as micromole of substrate converted per minute per milligram protein (*μ*mol/min/mg protein).

#### 2.6.5. Histochemical Observation of Glycogen Concentration

Tissue glycogen concentration was estimated histochemically, following Best′s method as described by Dawson [47]. Thin paraffin sections were dipped in xylene for 5 min then placed into absolute alcohol, and then in 1% (v/v) celloidin for 5 min, respectively. Then, the sections were stained with Ehrlich′s haemalum, and after washing in alcohol were later stained in Best′s carmine for 20 min, and embedded into Best′s diVerentiator for proper differentiation. The sections were dehydrated in absolute alcohol, cleared in xylene, and mounted in DPX. Photomicrographs were taken with a Leica DM6B microscope and K7 CMOS camera at 4X and 10X magnification.

#### 2.6.6. Histochemical Observation of Glycolytic Enzyme

HK, LDH, MDH, and G6PDH activities were assayed using cryostat sections of fresh frozen treated parasite tissues following the Pearse method [48]. The sections were incubated for 1 h at 37°C in their respective reaction media. The HK medium (per 10 mL) contained D‐glucose, NADP^+^, ATP, MgCl_2_, NBT, imidazole buffer, gelatin, and G6PDH. LDH was assayed in a 1:1 mixture of two solutions containing lactate, NBT, NaCN, PMS, polyvinyl alcohol, Tris buffer, and NAD. MDH and G6PDH media included NAD/NADP^+^, L‐malate, or glucose‐6‐phosphate, and a common NBT‐Tris‐MgCl_2_ stock (see details in Methods S1). Photomicrographs were taken with a Leica DM6B microscope and K7 CMOS camera at 4X and 10X magnification.

#### 2.6.7. Estimation of Nitric Oxide Synthase (NOS) Activity

NOS activity in treated parasite tissues was estimated following the Knowles and Salter method [49]. The reaction mixture contained 50 mM L‐arginine, 1.2 mM MgCl_2_, 0.24 mM CaCl_2_, 0.12 mM NADPH, and 50 mM potassium phosphate buffer (pH 7.2). To 900 *μ*L of this mixture, 100 *μ*L of tissue homogenate and 20 units of urease were added. The mixture was incubated at 37°C for 15 min. The reaction was terminated by adding 1 mL of 10% perchloric acid, and proteins were removed by centrifugation at 5000 rpm for 5 min. Citrulline production was measured at 490 nm using a UV‐Vis spectrophotometer.

#### 2.6.8. NO Estimation in the Treated Medium and Tissue

Live worms were incubated at 37°C for 2 h in petri dishes containing 10 mL of oxygen‐saturated treated medium. NO in this medium, lacking oxyhemoglobin, was oxidized primarily to nitrite (NO_2_^−^). NO_2_^−^ concentrations in both the medium and parasite tissue were measured following Sessa et al. [50]. One milliliter of incubating medium or 10% tissue homogenate was mixed with 1 mL of freshly prepared Griess reagent (equal parts of 0.5 g NED‐HCl and 0.5 g sulfanilamide, each dissolved in 50 mL distilled water). After 15 min of incubation at 37°C, absorbance was measured at 540 nm to quantify NO. A standard curve of sodium nitrite was prepared for the calculation of NO concentration.

### 2.7. Statistical Analysis

Statistical analysis was performed using GraphPad Prism 9.2. For comparison of more than two groups, one‐way ANOVA was performed. Data are shown as mean ± SD. Each experiment was conducted with *n* = 5. *p* < 0.05 was considered significant and *p* > 0.05 not significant. All enzyme assays were performed in triplicate for each sample.

## 3. Results

### 3.1. Effects on Glycogen Metabolism

Our result clearly indicates anthelmintic effects of senna plant extracts have a significant impact on glycogen catabolism in parasitic helminths. Biochemical analysis showed a notable decrease in glycogen levels: 96% with *S. alata*, 93% with *S. alexandrina*, and 89% with *S. occidentalis* compared to controls (Figure 1a). Furthermore, histochemical staining confirmed a similar reduction within all treated groups (Figure 1b). Moreover, GPase activity increased in all treated parasites, highest with *S. alata*, followed by *S. alexandrina* and *S. occidentalis*, with PZQ treatment showing the maximum increase (Figure 1c). Conversely, GSase activity significantly decreased in treated groups, with controls showing higher GSase activity (Figure 1d). These findings suggest that senna extracts disrupt parasite glycogen metabolism by depleting energy reserves. Increased glycogen breakdown (via GPase) and reduced synthesis (via GSase) limit ATP availability, impairing motility and survival, thereby contributing to anthelmintic effects.

Figure 1(a) The glycogen concentrations in both control and treated parasites exhibited a significant reduction across all treatments compared to the control group. (b) Histochemical micrographs of parasite cross‐sections showed reduced glycogen staining near the tegument in all treated groups compared to controls. (a,b) 4X magnification. (c,d) 10X magnification. (c) Effects of senna plant extracts on glycogen phosphorylase (GPase) enzyme activity and (d) glycogen synthase (GSase) enzyme activity in control and treated parasites demonstrated significant alterations in all treatment groups compared to the control. All data summarized the results from five rats with mean ± SD (*n* = 5). Significant difference was assessed by one‐way ANOVA and multiple comparison test, and the *p* values were considered significant at < 0.05 and marked as “∗”, *p* < 0.01 marked by “∗∗,” and *p* < 0.0001 marked by “∗∗∗∗” from control.

**Figure:**
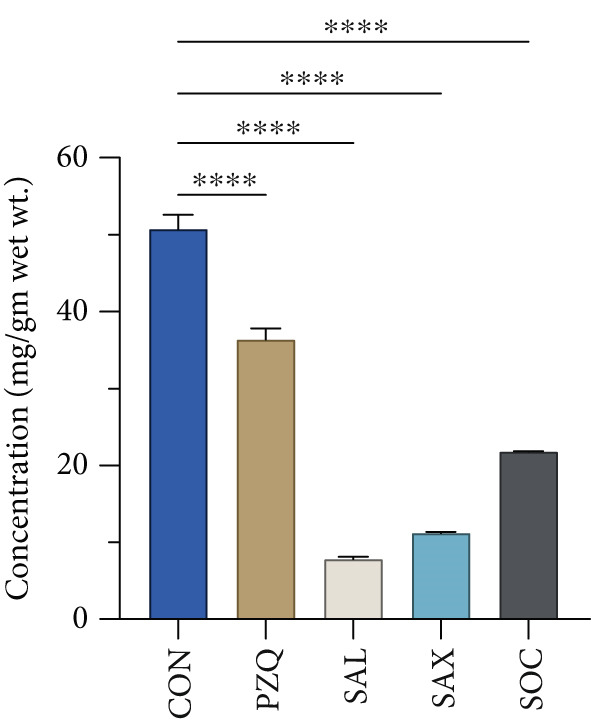
(a)

**Figure:**
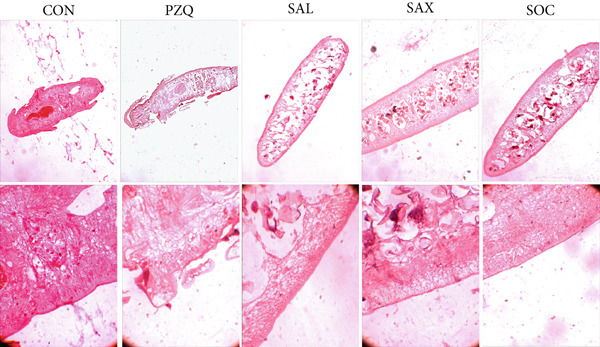
(b)

**Figure:**
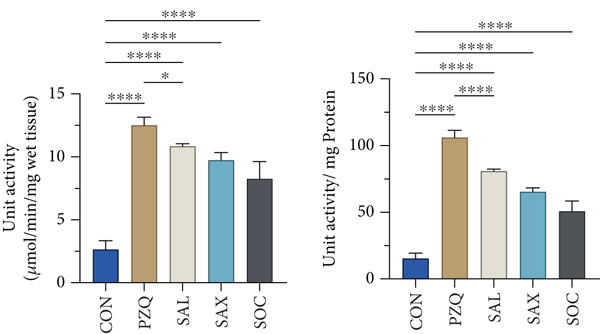
(c)

**Figure:**
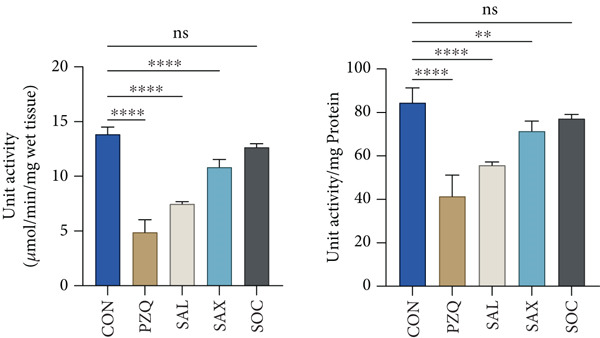
(d)

### 3.2. Glycolytic Metabolites

Treatment with senna plant extracts caused notable alterations in key glycolytic metabolites in parasitic helminths. Glucose levels increased significantly in all treated groups, with *S. alata* showing the highest rise, followed by *S*. *alexandrina*, *S*. *occidentalis*, and PZQ‐treated parasites (Figure 2a). In contrast, pyruvate and malate concentrations decreased substantially, with *S. alata* causing the most pronounced reductions, followed by *S*. *alexandrina, S*. *occidentalis*, and PZQ. *S. alata* caused the highest decrease around 68% in pyruvate, which is almost similar with PZQ (75%). Corresponding reductions of 49% and 36% were observed for *S. alexandrina* and *S. occidentalis*, respectively (Figure 2b). Malate levels declined markedly following treatment, with *S. alata* causing the greatest reduction (88%), followed by *S. occidentalis* (78%) and *S. alexandrina* (72%), whereas PZQ‐treated parasites showed a minimal decrease of 13% (Figure 2c) compared with control parasites. Furthermore, lactate levels significantly increased posttreatment, where control parasites showed 38.97 ± 1 *μ*mol/g wet tissue, whereas treated parasites had much higher levels: *S. alata* (201.24 ± 1.4 *μ*mol/g), *S. occidentalis* (164.47 ± 1.03 *μ*mol/g), and *S. alexandrina* (134.89 ± 1.3 *μ*mol/g) (Figure 2d). These results indicate that senna extracts disrupt parasite energy metabolism by creating a glycolytic bottleneck glucose accumulates, pyruvate and malate drop, and lactate rises reducing ATP production, impairing motility and contributing to parasite death.

Figure 2Effects of senna plants extracts in different metabolites concentration in control and treated parasites tissue of *Hymenolepis diminuta*. (a) Glucose, (b) pyruvate, (c) malate, and (d) lactate. All the data are reported as mean ± standard deviation (SD), post one‐way ANOVA analysis with *n* = 5 per group. Significance levels are denoted as follows: ∗∗∗∗ for *p* < 0.0001 and “ns” indicating nonsignificant results.

**Figure:**
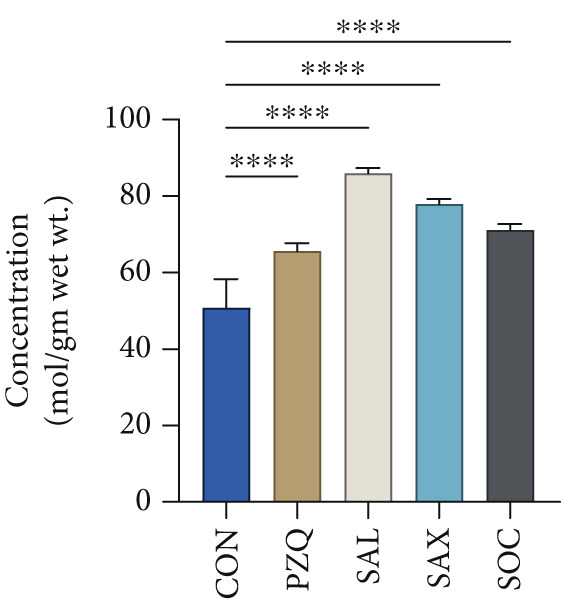
(a)

**Figure:**
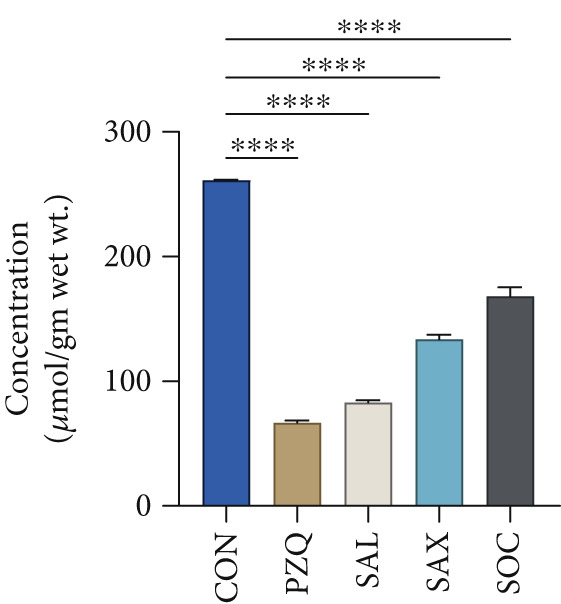
(b)

**Figure:**
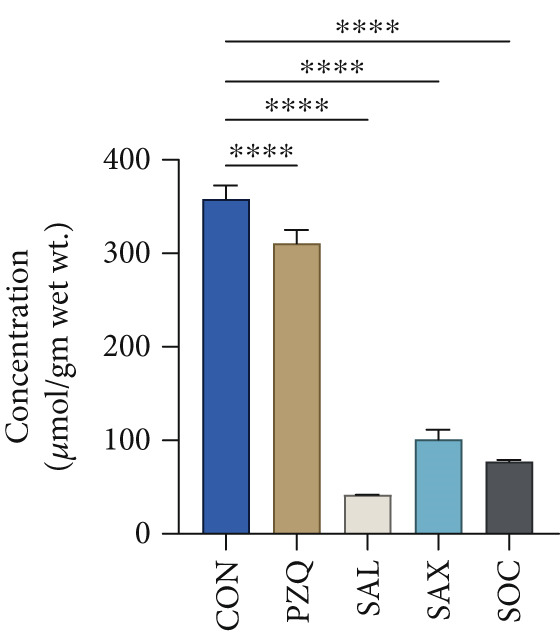
(c)

**Figure:**
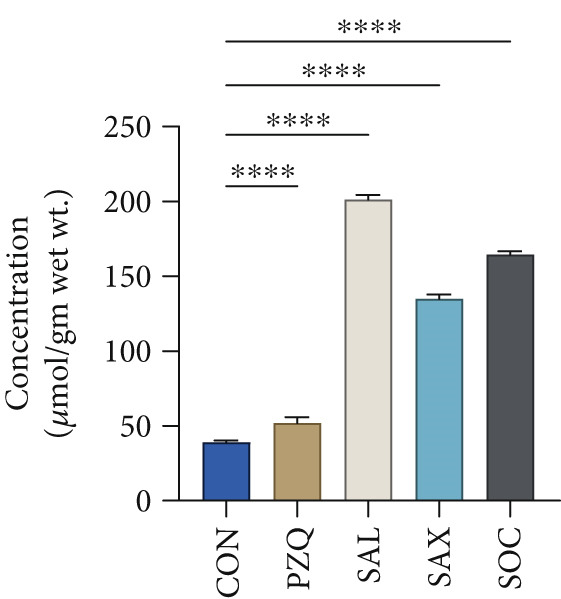
(d)

### 3.3. Effects on Glycolytic Enzymes Activity

The present study revealed that leaf extracts of three *Senna* species significantly altered the activities of key glycolytic enzymes in *H. diminuta*. HK activity increased substantially in all treated groups. Specific activity of HK rose by 63% in *S. alata*, 55% in *S. occidentalis*, 32% in *S. alexandrina*, and 184.6% in PZQ‐treated worms compared with the control group. Conversely, PFK activity decreased significantly by 55%, 45%, 40%, and 37%, respectively, across the same treatment groups. PK specific activity also declined, with 49%, 24%, and 33% reductions observed in *S. alata, S. alexandrina,* and *S. occidentalis*, respectively, whereas PZQ showed comparable inhibition. Similarly, PEPCK activity was inhibited by 40% in *S. alata*, 33% in *S. occidentalis*, and 15% in *S. alexandrina* (Table 1). LDH activity showed a significant rise in both crude homogenate and cytosolic fractions in all treated parasites. In crude homogenates, LDH increased by 100%, 62%, 54%, and 20% in *S. alata*, PZQ, *S. alexandrina*, and *S. occidentalis*, respectively. In the cytosolic fraction, the corresponding increases were 100%, 85%, 71%, and 71% (Table 2).

**Table 1:** Effects of three senna plant leaf extracts on unit activity (units/gram wet weight) and specific activity (units/milligram protein) of hexokinase (HK), phosphofructokinase (PFK), and pyruvate kinase (PK) of *Hymenolepis diminuta* in vitro.

**Treatments**	**Enzyme activity**							
	**HK**		**PFK**		**PK**		**PEPCK**	
	**Units activity**	**Specific activity**	**Units activity**	**Specific activity**	**Units activity**	**Specific activity**	**Units activity**	**Specific activity**
Control	4.71 ± 0.29^c^	0.65 ± 0.04^c^	10.98 ± 0.12^a^	1.5 ± 0.02^a^	68.55 ± 2.7^a^	7.45 ± 0.29^a^	42.5 ± 0.4^a^	4.36 ± 0.04^a^
PZQ	17.97 ± 1.47^a^	1.85 ± 0.15^a^	8 ± 0.18^b^	0.94 ± 0.02^b^	31.46 ± 0.29^e^	3.22 ± 0.03^e^	38.8 ± 0.3^b^	4.13 ± 0.03^b^
SAL	9.91 ± 0.34^b^	1.06 ± 0.04^b^	6.42 ± 0.32^d^	0.68 ± 0.04^d^	35.95 ± 0.26^d^	3.83 ± 0.03^d^	24.1 ± 1.1^e^	2.62 ± 0.12^e^
SOC	8.30 ± 0.54^b^	1.01 ± 0.08^b^	7.52 ± 0.1^c^	0.82 ± 0.01^c^	51.58 ± 0.69^b^	5.6 ± 0.08^b^	28.1 ± 0.5^d^	2.95 ± 0.1^d^
SAX	9.66 ± 0.73^b^	0.86 ± 0.08^b^	8.48 ± 0.18^b^	0.9 ± 0.02^b^	46.5 ± 0.4^c^	4.95 ± 0.04^c^	34.8 ± 0.5^c^	3.71 ± 0.06^c^

*Note:* Unit activity of enzyme: *μ*mol/min/g wet weight; specific activity: unit activity/mg protein. All the data are mean ± SE (*n* = 5). Different small alphabets indicate significant (*p* < 0.05) difference within a particular variable between values of different experimental groups following one‐way ANOVA and DMRT. One unit of enzyme activity is defined as that amount of enzyme which catalyzed 1 *μ*mol of NADP^+^ reduction (in the case of HK) or NADH oxidation (in the case of PFK, PK, and PEPCK) per minute at 38°C. Specific activity of the enzymes is expressed as the units of enzyme activity per milligram protein.

**Table 2:** Effects of three senna plants′ leaf extracts on unit activity (units/gram wet weight) and specific activity (units/milligram protein) of lactate dehydrogenase (LDH) and pyruvate carboxylase (PC) of *Hymenolepis diminuta* in vitro.

**Treatment**	**Enzyme activity**							
	**LDH**				**PC**			
	**Crude homogenate**		**Cytosolic fraction**		**Cytosolic fraction**		**Mitochondrial fraction**	
	**Unit activity**	**Specific activity**	**Unit activity**	**Specific activity**	**Unit activity**	**Specific activity**	**Unit activity**	**Specific activity**
Control	7.11 ± 0.34^e^	0.24 ± 0.01^e^	2.07 ± 0.15^c^	0.28 ± 0.02^c^	23.49 ± 0.26^a^	2.14 ± 0.02^a^	16.35 ± 0.8^a^	6.3 ± 0.3^a^
PZQ	12.22 ± 0.1^b^	0.39 ± 0.02^b^	4.1 ± 0.19^b^	0.52 ± 0.03^a^	21.09 ± 0.22^b^	1.96 ± 0.02^b^	11.85 ± 0.9^b^	4.24 ± 0.12^b^
SAL	13.74 ± 0.4^a^	0.48 ± 0.01^a^	5.67 ± 0.39^a^	0.56 ± 0.03^a^	16.88 ± 0.44^e^	1.68 ± 0.08^c^	8.61 ± 1.0^d^	2.41 ± 0.1^d^
SOC	10.1 ± 0.15^d^	0.29 ± 0.01^d^	4.31 ± 0.15^b^	0.48 ± 0.02^b^	19.07 ± 0.18^c^	1.89 ± 0.02^b^	9.76 ± 0.7^c^	3.86 ± 0.1^b^
SAX	11.1 ± 0.39^c^	0.37 ± 0.01^c^	4.47 ± 0.15^b^	0.48 ± 0.02^b^	18.6 ± 0.18^d^	1.78 ± 0.02^c^	9.17 ± 0.6^c^	3.18 ± 0.15^c^

*Note:* Unit activity of enzyme: *μ*mol/min/g wet weight; specific activity: unit activity/mg protein. All the data are mean ± SE (*n* = 5). Different small alphabets indicate significant (*p* < 0.05) difference within a particular variable between values of different experimental groups following one‐way ANOVA and DMRT. One unit of enzyme activity is defined as that amount of enzyme, which catalyzed 1 *μ*mol of NADH oxidation (in the case of LDH and PC) per minute at 38°C. Specific activity of the enzymes is expressed as the units of enzyme activity per milligram protein.

PC specific activity in the cytosolic fraction decreased by 22% (*S. alata*), 16% (*S. alexandrina*), 12% (*S. occidentalis*), and 8% (PZQ). In the mitochondrial fraction, PC activity dropped by 62%, 49%, 38%, and 33%, respectively. ME specific activity was also inhibited in both cytosolic and mitochondrial fractions: *S. alata, S. alexandrina,* and PZQ caused 61%, 25%, and 33% inhibition in the mitochondrial fraction, whereas cytosolic ME decreased by 68%, 66%, 63%, and 48%. No significant change was observed with *S*. *occidentalis* in the mitochondrial fraction (Table 3). Similarly, LDH and MDH activity increased by 43%, 46%, 17%, and 110% in crude homogenate; by 87%, 69%, 82%, and 101% in the mitochondrial fraction; and by 70%, 58%, 20%, and 103% in the cytosolic fraction of *S. alata, S. alexandrina, S. occidentalis,* and PZQ‐treated parasites, respectively (Table 4). The increase in HK alongside the decrease in PFK and PK suggests an initial accumulation of glucose‐6‐phosphate but a downstream bottleneck in glycolysis, reflecting an energy imbalance in the helminths. The observed elevations in LDH and MDH likely represent a compensatory response to maintain anaerobic metabolism under metabolic stress induced by senna extracts, while also contributing to the parasite′s stress response. Furthermore, the present study also revealed a significant increase in suppression of GDH activity in the cytosolic fraction of all treated worms; however in the mitochondrial fraction, GDH activity was suppressed significantly (Table 5). The G6PDH activity in the whole tissue homogenate of treated worms with *S. alata, S. alexandrina, S. occidentalis,* and PZQ decreased significantly (52%, 51%, 37%, and 59%) from control, respectively, in treatments with senna plant leaf extracts (Figure 3b), which correlates with the earlier reported downstream glycolytic bottleneck.

**Table 3:** Effects of three senna plants′ leaves extracts on unit activity (units/gram wet weight) and specific activity (units/milligram protein) of malic enzyme of *Hymenolepis diminuta* in vitro.

**Treatment**	**Enzyme activity**					
	**ME**					
	**Crude homogenate**		**Mitochondrial fraction**		**Cytosolic fraction**	
	**Unit activity**	**Specific activity**	**Unit activity**	**Specific activity**	**Unit activity**	**Specific activity**
Control	7.51 ± 0.15^d^	0.25 ± 0.01^c^	22.41 ± 0.38^a^	1.71 ± 0.02^a^	10.55 ± 0.57^a^	1.09 ± 0.06^a^
PZQ	11.5 ± 0.19^a^	0.42 ± 0.01^a^	12.62 ± 0.37^c^	1.15 ± 0.04^c^	5.35 ± 0.27^b^	0.57 ± 0.03^b^
SAL	9.91 ± 0.15^b^	0.29 ± 0.01^b^	6.87 ± 0.39^d^	0.66 ± 0.04^d^	2.56 ± 0.1^d^	0.35 ± 0.01^c^
SOC	8.31 ± 0.2^c^	0.24 ± 0.01^c^	18.86 ± 0.73^b^	1.7 ± 0.07^a^	3.43 ± 0.24^c^	0.37 ± 0.03^c^
SAX	8.15 ± 0.27^c^	0.28 ± 0.01^b^	12.86 ± 0.29^c^	1.28 ± 0.03^b^	3.76 ± 0.24^c^	0.4 ± 0.03^c^

*Note:* Unit activity of enzyme: *μ*mol/min/g wet weight; specific activity: unit activity/mg protein. All the data are mean ± SE (*n* = 5). Different small alphabets indicate significant (*p* < 0.05) difference within a particular variable between values of different experimental groups following one‐way ANOVA and DMRT. One unit of enzyme activity is defined as that amount of enzyme which catalyzed 1 *μ*mol of NADPH oxidation (in the case of ME) per minute at 38°C. Specific activity of the enzymes is expressed as the units of enzyme activity per milligram protein.

**Table 4:** Effects of three senna plants′ leaves extracts on unit activity (units/gram wet weight) and specific activity (units/milligram protein) of malate dehydrogenase (MDH) of *Hymenolepis diminuta* in vitro.

**Treatment**	**Enzyme activity**					
	**MDH**					
	**Crude homogenate**		**Mitochondrial fraction**		**Cytosolic fraction**	
	**Unit activity**	**Specific activity**	**Unit activity**	**Specific activity**	**Unit activity**	**Specific activity**
Control	8.47 ± 0.29^d^	0.28 ± 0.01^d^	6.63 ± 0.3^d^	0.63 ± 0.03^d^	7.51 ± 0.53^c^	0.82 ± 0.06^c^
PZQ	16.22 ± 0.51^a^	0.59 ± 0.02^a^	13.9 ± 0.32^a^	1.27 ± 0.03^a^	16.3 ± 1.23^a^	1.67 ± 0.13^a^
SAL	13.66 ± 0.51^b^	0.4 ± 0.02^b^	12.22 ± 0.48^b^	1.18 ± 0.02^b^	12.74 ± 0.65^b^	1.4 ± 0.02^b^
SOC	11.34 ± 0.24^c^	0.33 ± 0.01^c^	11.98 ± 0.22^b^	1.15 ± 0.02^b^	9.07 ± 0.56^c^	0.99 ± 0.06^c^
SAX	11.98 ± 0.22^b^	0.41 ± 0.01^b^	10.79 ± 0.25^c^	1.07 ± 0.03^c^	12.14 ± 0.1^b^	1.3 ± 0.01^b^

*Note:* Unit activity of enzyme: *μ*mol/min/g wet weight; specific activity: unit activity/mg protein. All the data are mean ± SE (*n* = 5). Different small alphabets indicate significant (*p* < 0.05) difference within a particular variable between values of different experimental groups following one‐way ANOVA and DMRT. One unit of enzyme activity is defined as that amount of enzyme which catalyzed 1 *μ*mol of NADH oxidation (in the case of MDH) per minute at 38°C. Specific activity of the enzymes is expressed as the units of enzyme activity per milligram protein.

**Table 5:** Effects of three senna plants′ leaves extracts on unit activity (units/gram wet weight) and specific activity (units/milligram protein) of glutamate dehydrogenase (GDH) of *Hymenolepis diminuta* in vitro.

**Treatment**	**Enzyme activity**					
	**GDH**					
	**Crude homogenate**		**Mitochondrial fraction**		**Cytosolic fraction**	
	**Unit activity**	**Specific activity**	**Unit activity**	**Specific activity**	**Unit activity**	**Specific activity**
Control	4.47 ± 0.23^d^	0.15 ± 0.01^c^	17.42 ± 1.0^a^	1.61 ± 0.09^a^	4.63 ± 0.24^e^	0.6 ± 0.03^c^
PZQ	7.75 ± 0.33^a^	0.27 ± 0.01^a^	13.98 ± 0.44^b^	1.43 ± 0.03^b^	8.1 ± 0.56^a^	0.96 ± 0.07^a^
SAL	6.13 ± 0.15^b^	0.19 ± 0.03^b^	10.39 ± 0.13^c^	0.96 ± 0.01^c^	7.65 ± 0.15^b^	0.81 ± 0.02^b^
SOC	5.11 ± 0.15^c^	0.15 ± 0.03^c^	8.95 ± 0.45^d^	0.86 ± 0.04^d^	6.15 ± 0.32^c^	0.65 ± 0.04^c^
SAX	5.67 ± 0.15^c^	0.2 ± 0.01^b^	8.95 ± 0.27^d^	0.89 ± 0.03^d^	5.51 ± 0.15^d^	0.6 ± 0.02^c^

*Note:* Unit activity of enzyme: *μ*mol/min/g wet weight; specific activity: unit activity/mg protein. All the data are mean ± SE (*n* = 5). Different small alphabets indicate significant (*p* < 0.05) difference within a particular variable between values of different experimental groups following one‐way ANOVA and DMRT. One unit of enzyme activity is defined as that amount of enzyme, which catalyzed 1 *μ*mol of NADH oxidation (in the case of GDH) per minute at 38°C. Specific activity of the enzymes is expressed as the units of enzyme activity per milligram protein.

Figure 3Effect of senna plants leaves extracts and reference drug Praziquantel *H. diminuta* on (a) histochemical micrographs of HK, LDH, MDH, and G6PDH activity in control and treated parasites of *H. diminuta*. Figures show cross‐sections through a mature proglottid. All figures are 10X magnification. (b) Effects of senna plants extracts on glucose 6‐phosphate dehydrogenase enzyme activity in control and treated parasites significant reduction occurred in all treatments in comparison to control. (c) NOS activity. (d) Efflux rate of NO in the incubation medium following exposure of the parasite into different senna plants leaf extracts. Results are presented as mean ± standard deviation (SD), following one‐way ANOVA with Sidak′s multiple comparisons test, *n* = 5 per group, where ∗∗∗ indicates *p* > 0.001, ∗∗∗∗*p* < 0.0001, and ns nonsignificant. All the photomicrograph at 10X magnification.

**Figure:**
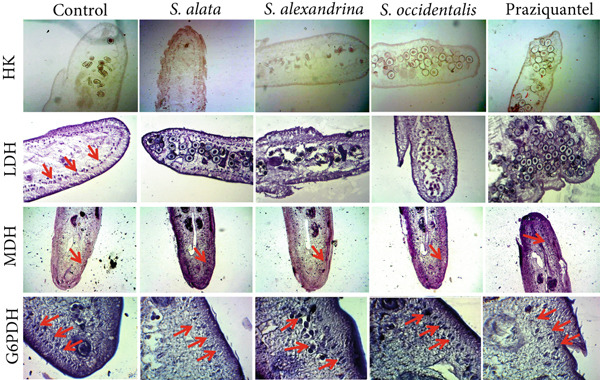
(a)

**Figure:**
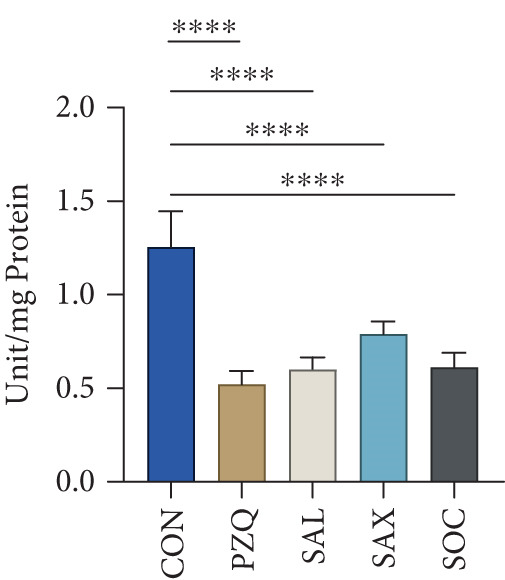
(b)

**Figure:**
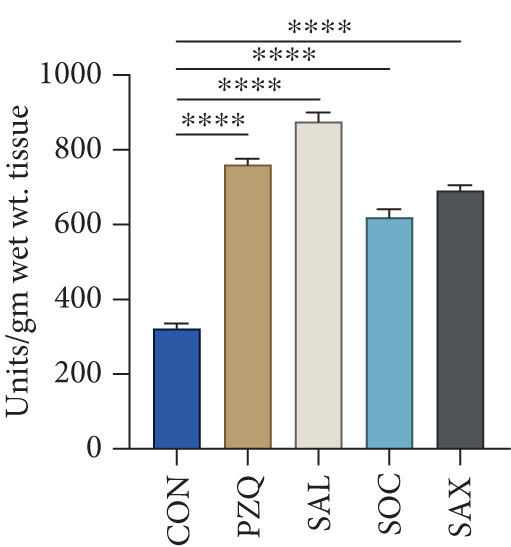
(c)

**Figure:**
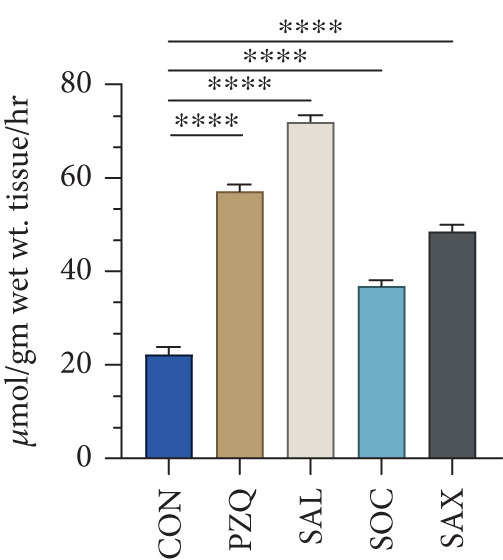
(d)

### 3.4. Enzymatic Histochemical Observation

Histochemical observations revealed noticeable alterations in the activity of HK, LDH, MDH, and G6PDH in the parasite tissue. Enhancements of the dark blue formazan granules were observed in the tissue section, indicating activities of the enzymes HK, LDH, and MDH, whereas reduction indicates the inhibition of enzymatic activity as found in G6PDH‐treated worms (Figure 3a). Histochemical changes indicate metabolic disruption in the parasites. Increased HK, LDH, and MDH reflect enhanced glycolysis and anaerobic compensation, whereas reduced G6PDH limits NADPH production. These alterations impair energy balance and stress management, reducing parasite survival.

### 3.5. Estimating NOS Enzymatic Activity

NOS activity was significantly elevated in all treated *H. diminuta* parasites compared to the control group (Figure 3c). Among the treatments, *S. alata* showed the highest NOS activity (879.56 ± 21.34 units/g wet wt. tissue), reflecting a 169.89% increase compared to control, followed by PZQ (134%), *S. alexandrina* (112%), and *S. occidentalis* (91%). Collectively, our results indicate that senna leaf extracts strongly upregulate the NOS pathway in parasites, leading to excessive NO production. This disrupts mitochondrial enzymes, damages proteins, and impairs neurotransmission, causing oxidative stress, energy imbalance, and neural dysfunction that ultimately weaken parasite survival and promote death.

### 3.6. Estimation of NO Efflux Into the Treated Medium

The increase in NOS activity in treated parasites was accompanied by a significant rise in NO efflux into the incubation medium (Figure 3d). Control parasites released 22.34 ± 1.36 *μ*mol/g wet wt. tissue/h of NO. Compared to controls, *S. alata*–treated parasites showed the highest increase (222%), followed by PZQ (157%), *S. alexandrina* (118%), and *S. occidentalis* (66%) (Figure 3d). These results suggest that both senna extracts and PZQ significantly stimulate NO release, potentially contributing to their anthelmintic effects.

## 4. Discussion

During glycolysis, several carbon atoms undergo oxidation, leading to the formation of pyruvate. The metabolic fate of pyruvate varies depending on the organism and its environmental conditions. In anaerobic environments, glucose is converted into two molecules of pyruvate, capturing a significant amount of energy [51]. In vertebrates, muscle tissues store three to four times more glycogen than the liver due to their greater mass [13]. During glucose scarcity, glycogen is broken down via glycogenolysis into glucose‐1‐phosphate, primarily by the action of GPase [52]. The amount of stored glycogen depends on several factors including environmental conditions, host feeding rate, physiological activity, and the number of parasites within the host at a given time [53–55].

Helminth parasites rely heavily on stored glycogen and simple carbohydrates like glucose for energy [56]. Among them, glycogen acts as the primary energy reserve in cestodes [1], with synthesis entirely dependent on host‐derived carbohydrates [4]. The current study demonstrated that the exposure of *H. diminuta* to senna leaf extracts resulted in approximately 90% depletion of glycogen. Histochemical analysis further revealed significantly reduced staining intensity in treated worms, indicating decreased glycogen content. These findings align with earlier reports in *Schistosoma* species and *Rallietina echinobothrida* treated with artemether and genistein [7, 57] [58]. The depletion of glycogen likely results from alterations in the activities of GPase and GSase. Treatment with three *Senna* species showed an increase in GPase activity and inhibition of GSase, indicating enhanced glycogenolysis and suppressed glycogenesis. This enzymatic shift suggests that stress induced by treatment increased the energy demand, leading to rapid glycogen breakdown. The corresponding rise in glucose concentration observed in *H. diminuta* treated with *S. alata, S. alexandrina*, and *S. occidentalis* supports this hypothesis. Elevated GPase activity under these conditions may have promoted glucose accumulation in the parasite tissues. A significant increase in glucose levels was recorded in treated parasites compared with the control parasites, further suggesting a stress‐induced metabolic response [59]. Similar patterns were reported in *Cotugnia digonopora* exposed to PZQ, niclosamide, and mebendazole [60]. Concomitant to this was a decrease in pyruvate and malate concentrations and an increase in lactate levels, suggesting a shift towards anaerobic metabolism. This implies that senna extracts may inhibit both glycolysis and the tricarboxylic acid (TCA) cycle in *H. diminuta*. Increased lactate production relative to malate may indicate a shift towards anaerobiosis, potentially driven by rapid muscular contractions, as previously noted in other cestodes [4]. Glycolytic enzymes are known to be regulated via allosteric modulation and covalent modifications [51]. Glycolysis is considered a promising drug target against parasites due to its crucial role in ATP production, as supported by studies on trypanosomatid parasites [61, 62]. Inhibiting glucose uptake or glycolytic enzymes can disrupt energy production, ultimately leading to parasite death. PZQ and mebendazole have been found to impair glucose uptake in several helminths [63], possibly through mitochondrial enzyme modulation [64]. Effective glycolysis inhibition may target key enzymes responsible for phosphorylating glycolytic intermediates, such as HK, PFK, PK, and PEPCK.

In the present study, HK activity increased significantly in all senna‐treated parasites, suggesting increased glucose utilization under stress conditions. This was also confirmed through histochemical analysis and agrees with findings by Das et al. [38]. Although HK is crucial for glycolytic entry, PFK acts as a central regulator of carbohydrate metabolism. In liver flukes, it is activated by fructose 2,6‐bisphosphate, and AMP [65–67]. However, in *Ascaris suum*, PFK is regulated through phosphorylation by specific kinases and inhibited by dephosphorylation [65, 68]. A significant decrease in PFK activity was noted in all treated worms in this study, indicating suppression of glycolysis due to senna exposure. Similar observations were previously reported in *Trypanosoma brucei* [69]. PEPCK, another critical regulatory enzyme, facilitates the conversion of oxaloacetate to phosphoenolpyruvate. Its activity was significantly inhibited in senna‐treated *H. diminuta*, echoing findings in *Haemonchus contortus* [70]. Similarly, PK activity was reduced in all treated samples, indicating that both PEPCK and PK, essential to energy production, are impaired. Such suppression aligns with previous observations in helminths treated with various anthelmintics [57] [71–74]. Pyruvate formed in glycolysis is converted to lactate by LDH, which also catalyzes the reverse reaction. In the present study, LDH activity increased significantly, which may be explained by the enhanced production of pyruvate from cytosolic ME activity. Elevated LDH activity, coupled with decreased pyruvate and increased lactate levels, reflects a metabolic adaptation to stress. Das et al. [38] also observed similar LDH behavior under drug‐induced stress.

MDH, another rate‐limiting enzyme in the phosphoenolpyruvate pathway, was also influenced by treatment. Although many drugs (e.g., oxyclozanide, hexachlorophene, and nitroxynil) inhibit MDH in trematodes [75], this study found a significant increase in MDH activity in all treated *H. diminuta* samples. This may reflect elevated malate levels within parasite tissues, aligning with findings by Das et al. [38]. The NAD‐dependent ME, responsible for converting pyruvate to L‐malate, showed increased activity in crude homogenates, mitochondria, and cytosolic fractions of *H. diminuta*. Similar enzyme activity has been noted in *Taenia crassiceps* larvae and various developmental stages of flukes [76, 77]. The HMP pathway, crucial for fatty acid synthesis and nucleic acid precursors, is regulated by G6PDH. Although G6PDH activity was high in control parasites, it was significantly reduced in senna‐treated worms. This suggests that NADPH synthesis was impaired under stress, a finding supported by studies in *Haemonchus contortus* [78]. PC, a crucial enzyme involved in gluconeogenesis and mitochondrial anaplerotic reactions, was significantly inhibited in senna‐treated *H*. *diminuta*, both in the cytosolic and mitochondrial fractions. The concurrent inhibition of PC and PEPCK suggests a strong suppression of gluconeogenic activity, consistent with previous reports on cestodes exposed to anthelmintic agents [70, 79, 80]. Therefore, increased HK activity with simultaneous decreases in PFK and PK indicates glucose‐6‐phosphate accumulation, whereas elevated LDH and MDH suggest compensatory maintenance of anaerobic metabolism. Reduced GDH and G6PDH activities likely exacerbate this metabolic imbalance by limiting NADPH regeneration and redox homeostasis, reinforcing energy stress. Collectively, these results support that senna extracts disrupt both glycolytic flux and ancillary pathways, causing metabolic stress and impairing energy production, which contributes to the parasite′s compromised viability. Furthermore, treated parasites exhibited a marked increase in NOS activity. This was supported by NADPH‐diaphorase staining, which revealed intense signals localized to the neck and scolex regions areas enriched in neuronal tissues. These findings are consistent with previous reports in *Fasciolopsis buski* and liver flukes treated with genistein or *Flemingia vestita* extracts. Elevated NOS activity by phytochemical constituents of senna leaf extracts led to excess NO production, triggering oxidative stress, DNA damage, calcium imbalance, and impaired neurotransmission [32, 81, 82]. These effects likely culminated in neuromuscular dysfunction and parasite death, highlighting the neurotoxic potential of senna phytochemicals. In summary, senna leaf extracts appear to disrupt multiple metabolic pathways in *H. diminuta*, including glycolysis, gluconeogenesis, the TCA cycle, and the HMP pathway. By modulating key enzymes and inducing oxidative and neurogenic stress, *Senna* spp. exerts a potent anthelmintic effect, providing a promising foundation for developing plant‐derived antiparasitic agents.

## 5. Conclusion

The study highlights that overuse of anthelmintic drugs leads to resistance, although economic limitations hinder the development of new treatments. Using metabolic pathway analysis, we identified novel targets for managing *H. diminuta*. Senna leaf extracts significantly increased NOS activity and NO efflux, triggering overproduction of NO in the parasite. This excess NO likely causes severe metabolic disruption by impairing mitochondrial enzymes, altering glycolytic flux, and disrupting neurotransmission. These changes lead to energy depletion, oxidative stress, neural paralysis, and ultimately, parasite death. Thus, the anthelmintic effects of *S*. *alata*, *S*. *occidentalis*, and *S*. *alexandrina* are mediated via metabolic and neurotoxic pathways, offering a promising strategy for new anthelmintic therapies.

## Ethics Statement

Protocols on animal handling and experimentation were followed strictly as per the guidelines of the Institutional Animal Ethics Committee (IAEC) Visva‐Bharati University and approval was obtained from IAEC bearing reference number Ref. No. IAEC/VB/2017/04.

## Disclosure

Funding sources were not involved in the study design, data collection, analysis, and interpretation. This work was carried out as part of the authors′ research activities at Visva‐Bharati University, West Bengal, India.

## Conflicts of Interest

The authors declare no conflicts of interest.

## Author Contributions

**Saptarshi Roy:** conceptualization, investigation, methodology, performed all the experiments, writing—original draft, and review and editing. **Larisha M. Lyndem:** conceptualization, methodology, writing—original draft, review and editing, and funding.

## Funding

This study was partially funded by the University Grants Commission, New Delhi, for providing financial assistance through a major research project (No. UGC/SR/40‐385/2011) sanctioned to L.M.L. The majority of the work is done by the UGC_BSR Fellowship awarded to S.R. [Ref. F.No. 25‐1/2014‐15(BSR)/5132/2007(BSR)].

## Supporting Information

Additional supporting information can be found online in the Supporting Information section.

## Supporting information


**Supporting Information 1** Methods S1: Detailed description of the biochemical experimental procedures, reaction mixture, and analytical methods used in this study.

**Supporting Information 2** Table S1: Detailed mortality times showing the effects of crude leaf extracts of senna plants and praziquantel on *Hymenolepis diminuta*.

## Data Availability

The data used to support the findings of this study are included in this article, and the original data can be requested from the corresponding author.
