# Supplementary material for: Exploring Metabolic Disruption and Redox Modulation by Senna Leaf Extracts Induces Mortality in the Zoonotic Parasite Hymenolepis diminuta
Source: J Parasitol Res. 2025 Dec 17;2025:2876272. doi: 10.1155/japr/2876272 (PMC12767092; doi:10.1155/japr/2876272)
Supplement: Supplementary file 1 — Supporting Information 1 Methods S1: Detailed description of the biochemical experimental procedures, reaction mixture, and analytical methods used in this study. [file JAPR-2025-2876272-s001.docx]

**Supplementary methods sections**

**S1. Intermediate host management and Cysticercoid development in *H. diminuta***

The intermediate host, Tribolium sp. beetles, was maintained on wheat flour supplemented with dried yeast under controlled laboratory conditions (28 ± 2 °C, 60–70% relative humidity). To establish infection, gravid proglottids from H. diminuta were collected from infected rats, homogenized in saline, and mixed with the beetle diet to allow ingestion of eggs. Infected beetles were separated after 24 hours and maintained for 3–4 weeks to permit cysticercoid development. Mature cysticercoids were dissected from beetles and orally administered to naïve Swiss albino rats using a gavage needle to complete the life cycle.

**S2. Details Methodes for all biochemical assays.**

*2.6. Biochemical Estimation*

*2.6.1. Estimation of glycogen*

Estimation of alkali soluble glycogen was measured by following the anthrone reagent method of Steifer et al [32]. Alkali-soluble glycogen was estimated using the anthrone reagent method described by Steifer et al. Approximately 100 mg of wet weight from treated and control parasite tissues was digested in 3 ml of 30% (w/v) KOH at 70°C for 20 minutes. After cooling, 0.2 ml of saturated Na₂SO₄ and 5 ml of 95% ethanol were added, followed by centrifugation at 10,000g for 10 minutes at 25°C to precipitate glycogen, with repeated three times. Glycogen was then quantified using 0.2% (w/v) anthrone in concentrated H₂SO₄, and absorbance was measured at 620 nm using a UV-VIS spectrophotometer. Concentrations were calculated using a standard curve.

*2.6.2. Estimation of different metabolites*

Glycolytic metabolites were estimated using the enzymatic method of Bergmeyer [33]. Treated and control parasites were homogenized (10% w/v) in 50 mM Tris–HCl buffer (pH 7.4), followed by the addition of 2 M perchloric acid (1:0.5) to precipitate proteins and macromolecules. After centrifugation at 10,000g for 10 minutes, the supernatant was collected and neutralized with 2 M NaOH. Metabolite levels were then assessed using specific reaction mixtures. For glucose, the reaction mixture contained 10 mM MgCl₂, 0.5 mM NADP⁺, 0.9 mM ATP, and 5 units each of glucose-6-phosphate dehydrogenase and hexokinase in 100 mM triethanolamine buffer (pH 7.6). For pyruvate, the mixture contained 0.2 mM NADH and 5 units of LDH in 50 mM Tris–HCl buffer (pH 7.4). L-lactate estimation involved glycine (0.5 mM), hydrazine hydrate (0.42 M), NAD⁺ (0.9 mM), and 5 units of LDH. For malate, the mixture included 100 mM Tris–HCl (pH 7.4), 1 mM glutamate, 1 mM NAD⁺, and 10 units each of malate dehydrogenase and glutamate oxaloacetate transaminase. Each mixture received 100 µl of tissue extract and was incubated at 38±1°C, 30 minutes for glucose, pyruvate, and malate, and 2 hours for lactate. Absorbance was measured at 340 nm using a UV-VIS spectrophotometer, and metabolite concentrations were calculated using a molar extinction coefficient of 6.22 × 10⁶ M⁻¹cm⁻¹ for NADH or NADPH.

*2.6.3. Estimation of Glycogen phosphorylase (GPase) and Glycogen synthase (GSase) activity.*

*2.6.3.1. Preparation of tissue sample*

A 10% (w/v) tissue homogenate of the treated and control parasites was prepared following the method of Russel and Storey [34] in homogenizing buffer, containing imidazole–HCl buffer (20mM, pH 7.2), NaF (100 mM), EDTA (10mM), EGTA (10mM), 2-mercaptoethanol (15mM) and PMSF (0.1mM). The homogenate was centrifuged at 10,000g at 4 °C for 10 min and the supernatant was used for the enzyme assays.

**GPase activity** was assayed following the method of Moon et al., [35]. The reaction mixture of 1 ml, contained 60mM potassium phosphate buffer (pH 7.2), NADP+ (0.5mM), glucose 1,6-bisphosphate (5mM), AMP (2.5mM), phosphoglucomutase (5 unit), G6PDH (5 unit), glycogen (10 mg) and tissue extract (100 µl). And for **GSase activity** assayed following the method of Passoneau and Rottenberg [36]. The reaction mixture contained imidazole–HCl buffer (60mM, pH 7.5), phosphoenolpyruvate (5mM), UDP-glucose (6mM), NADH (0.15mM), KCl (150mM), MgCl_2_ (15mM), glycogen (2mg), PK (10Unit), LDH (10Unit), 5 µmol glucose 6-phosphate. A 100µl of tissue extract was added to the preincubated assay mixture at 38 °C for 10min.

*2.6.4. Glycolytic Enzyme Assay*

*2.6.4.1. Tissue processing*

To assay key regulatory glycolytic and carbohydrate metabolism enzymes, tissue processing followed the method of Das et al. [37]. Frozen tissues were thawed on ice, and 10% (w/v) homogenates were prepared in buffer containing 50 mM Tris-HCl (pH 7.4), 0.3 M sucrose, 1 mM EDTA, 2 mM MgCl₂, and 3 mM 2-mercaptoethanol. The homogenates were treated with 0.5% Triton X-100 (1:1) for 30 minutes, followed by 30 seconds of sonication (Soniprep 150) to disrupt mitochondria. The samples were then centrifuged at 10,000g for 15 minutes at 4°C, and the supernatant was used for enzyme assays.

*2.6.4.2. Sub-cellular fraction*

Mitochondrial and cytosolic fractions were prepared by following the methods of Das et al., [37]. The differential centrifugation of a 10% homogenate of the parasite tissue in a fractionating buffer containing 50 mM Tris-HCl (pH 7.4), 0.3 M sucrose, 1 mM EDTA, 2 mM MgCl_2_ and 3 mM 2-mercaptoethanol. LDH was used as the cytosolic marker and glutamate dehydrogenase [GDH; L-glutamate: NAD(P)+ oxidoreductase; EC 1.4.1.3] as the mitochondrial marker to access the complete separation process of the different sub-cellular fractions.

*2.6.4.3. Estimation of enzymes activity*

The activity of key glycolytic and carbohydrate metabolism enzymes was measured using standard spectrophotometrically. Hexokinase activity was assayed following Bergmeyer [35] using a 1 ml reaction mixture containing 50 µmol Tris-HCl buffer (pH 7.4), 5 µmol D-glucose, 0.2 µmol NADP⁺, 0.9 µmol ATP, 5 µmol MgCl₂, 10 units of glucose-6-phosphate dehydrogenase, and 100 µl of tissue extract. Phosphofructokinase activity followed the modified method of Buckwitz et al. [38] with a reaction mixture comprising 80 µmol Tris-HCl (pH 7.2), 5 µmol fructose-6-phosphate, 0.2 µmol NADH, 0.8 µmol ATP, 0.9 µmol MgCl₂, 0.9 µmol KCl, 1 µmol K₂HPO₄, 8 µg aldolase, 3.3 µg triosephosphate isomerase, 3.3 µg glycerophosphate dehydrogenase, and 100 µl of extract. Phosphoenolpyruvate carboxykinase activity was assayed using the method of Mommsen et al. [39] with a mixture containing 50 µmol Tris-HCl (pH 7.4), 4.5 µmol phosphoenolpyruvate, 0.15 µmol NADH, 0.6 µmol GDP, 20 µmol NaHCO₃, 1 µmol MnCl₂, 5 units MDH, and tissue extract. Pyruvate kinase was assayed using Bucher and Pfleiderer [40], using imidazole buffer (pH 7.6), phosphoenolpyruvate, NADH, ADP, KCl, MgSO₄, LDH, and extract. LDH activity followed Vorhaben and Campbell [41], using sodium phosphate buffer, pyruvate, NADH, and extract. MDH activity was measured following Kun and Volfin [42] using oxaloacetate and NADH. Malic enzyme activity followed Bergmeyer [35], using Tris-HCl, pyruvate, NADPH, and NaHCO₃. Pyruvate carboxylase activity was estimated by the Moon and Mommsen [39] method using Tris-HCl (pH 7.8), acetyl CoA, NaHCO₃, pyruvate, NADH, MgCl₂, MDH, and extract. G6PDH activity was estimated using DeMoss [43] with Tris-HCl, MgCl₂, NADP⁺, and glucose-6-phosphate. Glutamate dehydrogenase activity was measured using Olson and Anfinsen [44], with potassium phosphate buffer (pH 8.5), ammonium chloride, α-ketoglutarate, NADH, and EDTA. All assays included 100 µl of tissue extract. The enzymes activities were estimated using pre-incubated assay mixture at 38°C for 5 min. then add tissue extract to start the reaction. OD was recorded at 340 nm at 10 sec intervals for 3 min by using a UV-Visible spectrophotometer. Enzyme specific activity was expressed as Unit/mg of protein.

*2.6.6. Histochemical observation of Glycogen concentration*

Tissue glycogen concentration was estimated histochemically, following Best’s method as described by Dawson [45]. Thin paraffin sections were dipped in xylene for 5 min then placed into absolute alcohol then in 1% (v/v) celloidin for 5 min respectively. Then sections were stained with Ehrlich’s haemalum and after washing in alcohol, were later stained in Best’s carmine for 20min, and embedded into Best’s diVerentiator for proper differentiation. The sections were dehydrated in absolute alcohol, cleared in xylene and mounted in DPX.

*2.6.7. Histochemical observation of Glycolytic enzyme*

HK, LDH, MDH and G6PDH activities was performed using cryostat sections of fresh frozen treated parasite tissues, following standard procedures by Pearse [46]. Sections were incubated for 1 hour at 37°C in their respective reaction media. The HK incubation medium (per 10 ml) contained 30 mg D-glucose, 2.5 mg NADP⁺, 5.5 mg ATP, 20 mg MgCl₂, 2.5 mg NBT, 2 ml 40 mM imidazole buffer (pH 7.5), 3.8 ml 6% gelatin, and 5 µl G6PDH. LDH activity was assayed in a 1:1 mixture of solution A (L-lactate, NBT, NaCN, PMS, polyvinyl alcohol in Tris buffer) and solution B (polyvinyl alcohol in Tris buffer) with 1.5 mM NAD. MDH medium contained NAD, neutralized L-malate, and a stock solution with NBT, Tris-HCl buffer, MgCl₂, and water. G6PDH medium contained NADP⁺, glucose-6-phosphate, acidified with HCl, and the same stock solution as for MDH. All the picture was taken by light microscope.

*2.6.8. Estimation of Nitric Oxide Synthase (NOS) activity*

NOS activity in treated parasite tissues was estimated following Salter and Knowles [47]. The reaction mixture contained 50 mM L-arginine, 1.2 mM MgCl₂, 0.24 mM CaCl₂, 0.12 mM NADPH, and 50 mM potassium phosphate buffer (pH 7.2). To 900 µl of this mixture, 100 µl of tissue homogenate and 20 units of urease were added. The mixture was incubated at 37°C for 15 minutes. The reaction was terminated by adding 1 ml of 10% perchloric acid, and proteins were removed by centrifugation at 5000 rpm for 5 minutes. Citrulline production was measured at 490 nm using a UV-VIS spectrophotometer.

*2.6.9. NO estimation in the treated medium and tissue*

Live worms were incubated at 37°C for 2 hours in petri dishes containing 10 ml of oxygen-saturated treated medium. Nitric oxide in this medium, lacking oxyhemoglobin, was oxidized primarily to nitrite (NO₂⁻). NO₂⁻ concentrations in both the medium and parasite tissue were measured following Sessa et al. [48]. One milliliter of incubating medium or 10% tissue homogenate was mixed with 1 ml of freshly prepared Griess reagent (equal parts of 0.5 g NED-HCl and 0.5 g sulfanilamide, each dissolved in 50 ml distilled water). After 15 minutes of incubation at 37°C, absorbance was measured at 540 nm to quantify NO. A standard curve of sodium nitrite was prepared for the calculation of NO concentration.
