# Supplementary material for: Exploring Metabolic Disruption and Redox Modulation by Senna Leaf Extracts Induces Mortality in the Zoonotic Parasite Hymenolepis diminuta
Source: J Parasitol Res. 2025 Dec 17;2025:2876272. doi: 10.1155/japr/2876272 (PMC12767092; doi:10.1155/japr/2876272)
Supplement: Supplementary file 2 — Supporting Information 2 Table S1: Detailed mortality times showing the effects of crude leaf extracts of senna plants and praziquantel on Hymenolepis diminuta. [file JAPR-2025-2876272-s002.doc]

**Supplementary Material**

**Supplementary Table-1. Effects of crude leaf extract of Senna plants and Praziquantel on Hymenolepis diminuta, (Adopted from Kundu et al, 2012).**

| Treatment | PT(hr) | TM(hr) |
| --- | --- | --- |
| Control | - | 69.22±0.23 |
| Praziquental | 0.31±0.01 | 17.31±0.13 |
| *S. alata* | 1.68±0.06 | 9.39±0.14 |
| *S. alexandrina* | 2.32±0.1 | 16.65±0.3 |
| *S. occidentalis* | 3.86±0.10 | 20.42±0.21 |
| Each value represented as mean ± SE (no=6),PT=paralysis time (hours), TM= time of mortality (hours) | | |
